# In This Issue: Volume 116, Issue 4, April 2025

**DOI:** 10.1111/cas.70053

**Published:** 2025-04-03

## 1 GD2 Is a Crucial Ganglioside in the Signal Modulation and Application as a Target of Cancer Therapeutics

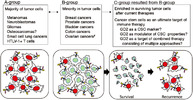

Gangliosides are complex fat‐sugar molecules found in the membranes of both healthy and cancerous cells. Among them, GD2 and its precursor, GD3, are present in high amounts on cancer cells, making them key targets for cancer immunotherapy. Understanding their roles could help refine targeted treatments and improve patient outcomes.

In this study, Furukawa et al. reviewed how GD2 and GD3 contribute to tumor development across various cancers. They found that GD3 is primarily involved in early tumor growth, while GD2 plays a key role in metastasis and adhesion to the extracellular matrix. High levels of GD2 were observed in melanoma, lung cancer, adenocarcinoma (gland cancer), and osteosarcoma—a type of bone cancer. Additionally, GD2 and GD3 were detected in gliomas and other cancers of neural origin, where they were linked to increased tumor malignancy. Notably, GD2 was identified as a marker of breast cancer stem cells, which are linked to tumor initiation and therapy resistance.

The study also examined how gangliosides interact with proteins to promote cancer progression. GD3 was linked to neogenin‐1, a receptor that enhances malignancy, while GD2 interacted with integrin β1 in melanoma and ASCT2, a glutamine transporter, in small cell lung cancer. These interactions help cancer cells survive, invade surrounding tissues, and resist treatment.

Because GD2 is mostly absent from normal tissues, it has been a major focus of immunotherapy. Anti‐GD2 monoclonal antibodies have been used for decades in melanoma and neuroblastoma, and more recently, CAR T‐cell therapy has emerged as a promising treatment. However, challenges remain, particularly in solid tumors such as prostate, bladder, and colon cancer, where only some cancer cells express GD2, suggesting GD2‐positive cells to be cancer stem‐like cells and potentially escape treatment.

These findings highlighted the promise of GD2‐targeted therapies while emphasizing the need for combination approaches to improve treatment effectiveness. The authors suggest that integrating GD2‐targeted therapies with other cancer treatments could lead to better patient outcomes. Further research is needed to determine whether GD2‐targeted immunotherapy should be prioritized for treatment‐resistant cancers, paving the way for more effective treatment strategies.

https://onlinelibrary.wiley.com/doi/10.1111/cas.70011

## 2 Mutation Analysis of TMB‐High Colorectal Cancer: Insights Into Molecular Pathways and Clinical Implications

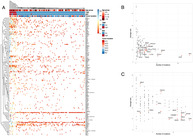

Colorectal cancer (CRC) is one of the most well‐studied cancers in terms of genetic mutations. Mutations in the *APC*, *TP53*, and *KRAS* genes are recognized as some of the key drivers of tumor development. Not all CRCs, however, follow this traditional genetic pathway. Certain tumors, especially those located on the right side of the colon, show a high tumor mutation burden (TMB) and appear to follow a different mechanism of tumor development.

In this study, Chikaishi et al. explored the distinct molecular characteristics of these high‐TMB tumors and their potential treatment implications. By analyzing targeted sequencing data, the researchers reported that unlike typical CRCs, high‐TMB tumors have fewer *APC* and *KRAS* gene mutations and instead display a higher chance of mutations in the *BRAF* gene. They also noted that mutations in genes involved in DNA repair (like *ATM* and *POLE* genes) often appear early in the development of these tumors, suggesting that impairment of these pathways might be an important event in their development.

The study also found that TMB‐high tumors often had mutations in pathways that regulate cell growth, such as the RTK‐RAS and PI3K pathways which, in turn, enabled the growth and survival of the cancer cells. The presence of high TMB in these tumors suggests that they may respond better to immune checkpoint inhibitors, a type of cancer treatment that helps the immune system recognize and attack cancer cells.

Overall, the study revealed that CRC with a high TMB is a distinct subgroup of the disease, with unique genetic features and pathways. Since these tumors seem less dependent on the common mutations (like *APC*, *TP53*, and *KRAS*) and are more genetically diverse, they might be a good target for immunotherapy.

https://onlinelibrary.wiley.com/doi/10.1111/cas.16455

## 3 Transplantation of the *MSLN*‐Deficient Thymus Generates MSLN Epitope Reactive T Cells to Attenuate Tumor Progression

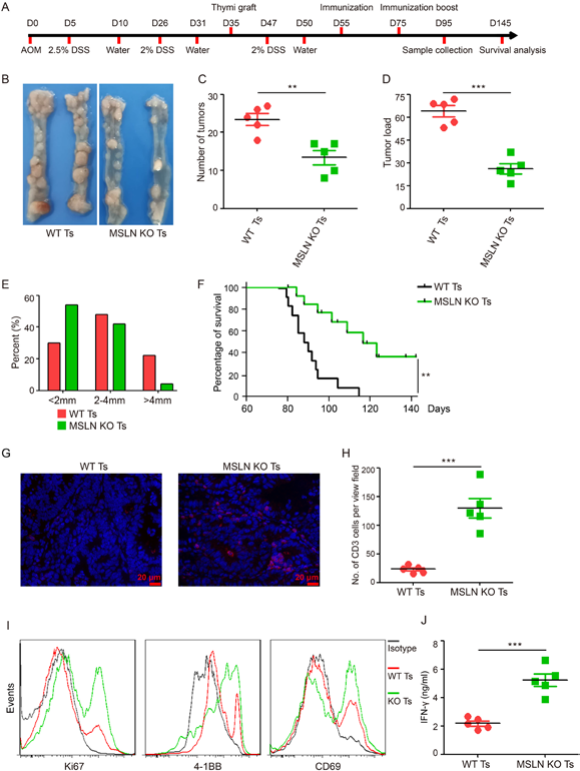

Mesothelin (MSLN) is a protein that is highly expressed in several cancers, making it a promising target for immunotherapy. However, because MSLN is also expressed in healthy tissues at low levels, the immune system often tolerates it, limiting the generation of MSLN‐specific T cells that can effectively target tumors. This study explores a novel approach to overcoming this limitation by transplanting MSLN‐deficient thymus tissue into mice, allowing the development of reactive T cells capable of targeting MSLN‐expressing tumors.

The researchers demonstrated that mice receiving MSLN‐deficient thymus transplants naturally generated MSLN‐reactive T cells. These T cells infiltrated tumors and significantly impeded cancer progression without causing harmful autoimmune responses. Compared to conventional chimeric antigen receptor (CAR) T‐cell therapy, which involves modifying T cells outside the body and reintroducing them, this approach resulted in stronger and more sustained immune responses. CAR‐T cells often lose efficacy due to tumor immune escape mechanisms or prolonged ex vivo manipulation, whereas the T cells generated through thymic transplantation remained effective over time.

The study further validated the approach in an AOM‐DSS‐induced colorectal cancer (CRC) mouse model, where transplantation of MSLN‐deficient thymus significantly reduced tumor progression and increased the survival rates. The treated mice exhibited higher levels of IFNγ‐expressing T lymphocytes within tumors, indicating a robust anti‐tumor immune response. Moreover, transcriptome analysis of tumor‐infiltrating immune cells revealed enhanced T‐cell proliferation and cytotoxicity, suggesting that the MSLN‐deficient thymus empowers the immune system by generating highly functional tumor‐targeting T cells.

These findings introduce a promising new strategy for cancer immunotherapy, leveraging the body's thymus to generate tumor‐specific T cells naturally. Unlike current CAR‐T therapies, which face challenges such as tumor escape mechanisms and severe toxicities, this approach offers a potentially more controlled and long‐lasting immune response. Future research could explore the development of thymic organoids with specific antigen deletions to further refine this method and expand its clinical applications.

https://onlinelibrary.wiley.com/doi/10.1111/cas.16458

